# Diabetic Retinal and Choroidal Edema in SDT Rats

**DOI:** 10.1155/2016/2345141

**Published:** 2015-12-13

**Authors:** Fumihiko Toyoda, Yoshiaki Tanaka, Machiko Shimmura, Nozomi Kinoshita, Hiroko Takano, Akihiro Kakehashi

**Affiliations:** Department of Ophthalmology, Jichi Medical University, Saitama Medical Center, 1-847 Amanuma-cho, Omiya-ku, Saitama, Saitama 330-8503, Japan

## Abstract

We evaluated the features of diabetic retinal and choroidal edema in Spontaneously Diabetic Torii (SDT) rats. We measured the retinal and choroidal thicknesses in normal Sprague-Dawley (SD) rats (*n* = 9) and SDT rats (*n* = 8). The eyes were enucleated 40 weeks later after they were diagnosed with diabetes, and 4-micron sections were cut for conventional histopathologic studies. The mean retinal and choroidal thicknesses were significantly thicker in the SDT rats than in the normal SD rats. The choroidal thickness was correlated strongly with the retinal thickness in both rat models. Diabetic retinopathy (DR) and diabetic choroidopathy appeared as edema in the SDT rats. The retinal thickness was correlated strongly with the choroidal thickness in the SDT rats, which is an ideal animal model of both DR and choroidopathy.

## 1. Introduction 

Diabetic retinopathy (DR) is a major cause of visual loss and blindness in developed countries [1]. Physicians need to understand the manner in which DR develops and how it can be prevented by using animal models of diabetes. With that in mind, an animal model of diabetes with ocular complications mimicking human diabetics is needed. Many diabetic animal models have been reported. Énzsöly et al. [2] reported that degenerative changes in the photoreceptors and pigment epithelium appeared in streptozotocin-induced diabetic rats. Those investigators used male Wistar and Sprague-Dawley (SD) rats and found no significant difference between the retinal thicknesses in the normal and diabetic rats. Long-Evans Tokushima Learn rats have been used as a model of type 1 diabetes [3, 4]. Pancreatic changes and genetic analysis were discussed in those studies, but ocular complications were not mentioned. Otsuka Long-Evans Tokushima Fatty (OLETF) rats are a well-known model of type 2 diabetes. Using spectral-domain optical coherence tomography (OCT), Yang et al. [5] reported that the retinas were significantly thinner in OLETF rats than in normal Long-Evans Tokushima Otsuka rats and the tendency was apparent in the retinal nerve fiber layer (NFL). Diabetic animal models and their ocular changes in these studies are important to the understanding of diabetic ocular complications. However, the ocular findings in these models differ from those in humans.

A new spontaneously diabetic strain of the SD rat, the Spontaneously Diabetic Torii (SDT) rat, was established in 1997 and the features of the model were reported [6]. Mature diabetic cataracts and proliferative DR (PDR) especially resemble human diseases in SDT rats. These remarkable ocular complications do not appear in any other animal models of diabetes. We think that SDT rats are the most ideal model of diabetic ocular complications, and we used them to examine the effect of ranirestat, a new aldose reductase inhibitor, on DR [7].

Although several interesting studies [8–11] have been published about the relationship between the retina and choroid in patients with diabetic macular edema (DME), to the best of our knowledge no study has reported the choroidal thickness in diabetic model rats. In the current study, we evaluated the retinal and choroidal edema in SDT rats.

## 2. Materials and Methods

### 2.1. Animals

The care and handling of animals were in accordance with the Association for Research in Vision and Ophthalmology Statement for the Use of Animals in Ophthalmic and Vision Research and the Jichi Medical University Animal Care and Use Committee. We obtained male SDT rats and normal SD rats from CLEA, Inc. (Tokyo, Japan). All SDT rats (*n* = 8) were confirmed to be diabetic based on a nonfasting blood glucose concentration exceeding 350 mg/dL. The SDT rats were diagnosed with diabetes by 12 to 20 weeks after birth. All SDT rats were fed standard rat chow (CRF-1, Oriental Yeast, Inc., Tokyo, Japan) for 40 weeks after the onset of diabetes. All normal SD rats (*n* = 9) were fed the same rat chow as SDT rats. All SDT rats and SD rats were over 50 weeks old.

### 2.2. Measurement of Body Weight, Blood Glucose, and Glycated Hemoglobin

Body weight, blood glucose, and glycated hemoglobin (HbA1c) were measured once monthly. Blood samples to measure the blood glucose and HbA1c were collected from the tail vein of nonfasting rats. Blood glucose was measured by the hexokinase/glucose-6-phosphate dehydrogenase method (L type Wako Glu2, Wako Pure Chemical Industries, Ltd., Osaka, Japan). HbA1c was measured using an automated glycohemoglobin analyzer (HLC-723GHb V, Tosoh Corporation, Tokyo, Japan).

### 2.3. Ocular Histopathology

Some ocular histopathology procedures were the same as the methods we reported previously [7]. Under deep anesthesia induced by an intraperitoneal injection of pentobarbital sodium (25 mg/kg body weight, Nembutal, Sumitomo Dainippon Pharmaceutical Co., Ltd., Osaka, Japan), the eyes were enucleated for conventional histopathologic studies and placed in a fixative (Super Fix KY-500, Kurabo, Japan). The fixed eyes were washed in 0.1% mol/L cacodylate buffer and embedded in paraffin. The paraffin block was sectioned to 4 *μ*m and stained with hematoxylin and eosin for conventional histopathologic examination.

### 2.4. Measurement of Retinal and Choroidal Thicknesses

The 4-*μ*m paraffin blocks were examined using a polarizing microscope (Olympus BX-51, Olympus Corporation, Tokyo, Japan), and the images were recorded and downloaded using the attached digital camera and software (Olympus DP 72, DP2-BSW, Olympus Corporation). The retinal thickness was defined as the distance between the retinal internal limiting membrane (ILM) and the retinal pigment epithelium (RPE). The choroidal thickness was defined as the distance between the RPE and the choroidal-scleral junction. In the retina, the thicknesses between the ILM and the inner nuclear layer (INL), the INL thickness, the outer nuclear layer (ONL) thickness, and the photoreceptor layer (PL) thickness were calculated. The mean retinal and choroidal thicknesses were measured 500, 1,000, and 1,500 microns from the optic nerve disc using ImageJ software (National Institutes of Health, Bethesda, MD, USA).

### 2.5. Statistical Analysis

All values were expressed as the mean ± standard deviation. The Mann-Whitney* U* test was used for comparisons between two groups. Spearman's rank-order correlation was used to evaluate the relationship between retinal and choroidal thicknesses. Excel Tokei 2006 software (the Social Survey Research Information Co., Ltd., Tokyo, Japan) was used for statistical analysis. *P* < 0.05 was considered statistically significant.

## 3. Results

### 3.1. Body Weight, Blood Glucose, and Glycated Hemoglobin

**Figure 1:**
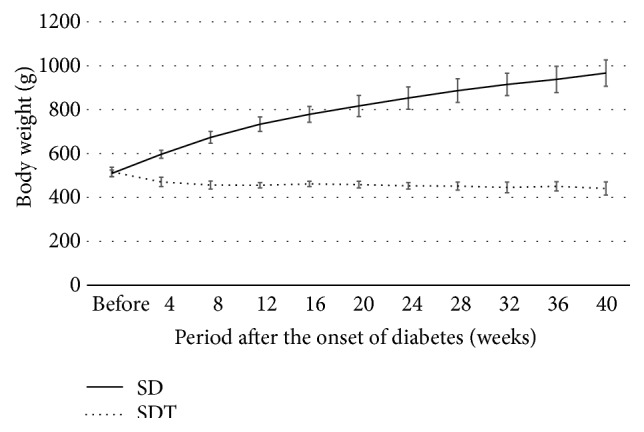
Body weight of the study animals. The SD rats are heavier than the SDT rats.

**Figure 2:**
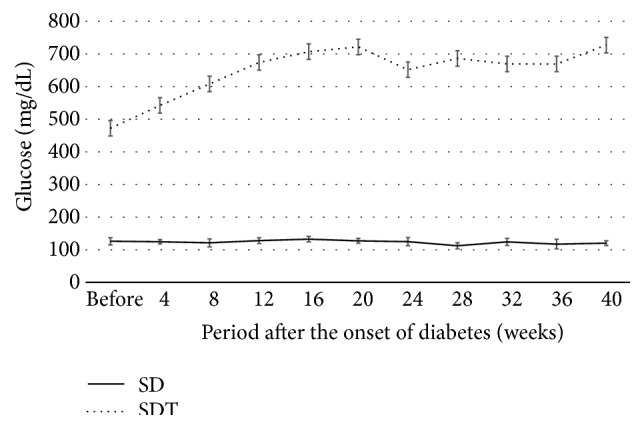
Blood glucose levels of the study animals. The mean blood glucose levels of the SD rats are significantly lower than those of the SDT rats.

**Figure 3:**
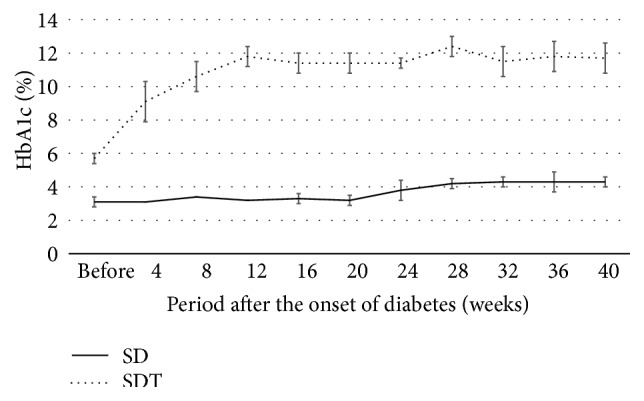
HbA1c levels of the study animals. The mean HbA1c levels of the SD rats are significantly lower than those of the SDT rats.

Figures 1, 2, and 3 show the changes in weight, blood glucose, and HbA1c, respectively, during the study. Compared with the SD rats, the SDT rats were significantly (*P* < 0.01) lighter. The mean blood glucose levels and HbA1c levels of the SDT rats were significantly (*P* < 0.01) higher than those of the SD rats.

### 3.2. Retinal and Choroidal Thicknesses

**Figure 4:**
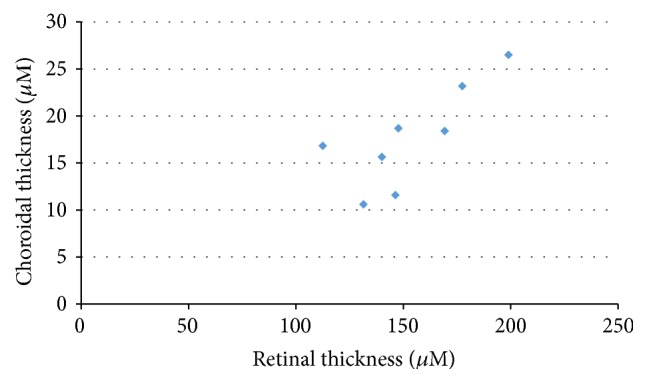
The relationship between the retinal and choroidal thicknesses in the SDT rats. *r*_*s*_ = 0.81, *P* < 0.05.

**Figure 5:**
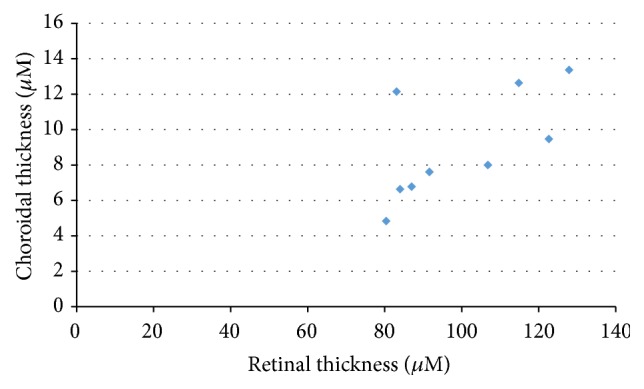
The relationship between the retinal and choroidal thicknesses in the normal SD rats. *r*_*s*_ = 0.72, *P* < 0.05.

Tables 1 and 2 show the retinal and choroidal thicknesses in the normal SD rats and the SDT rats, respectively. The mean values are shown based on the distance from the optic nerve disc (500, 1,000, and 1,500 *μ*m), and the average of three values was calculated and shown. Most of the retinas and choroids were significantly thicker in the SDT rats than in the normal SD rats, but there were no significant differences in the INL and choroidal thicknesses 500 *μ*m from the optic nerve disc. Figures 4 and 5 show the relationship between the retinal and choroidal thicknesses (average of three measurement points) in the SDT rats and the normal SD rats, respectively. The choroidal thicknesses were correlated strongly with the retinal thicknesses in the SDT rats and the normal SD rats (*r*_*s*_ = 0.81, *P* < 0.05, and *r*_*s*_ = 0.72, *P* < 0.05, resp.).

### 3.3. Histopathologic Studies

**Figure 6:**
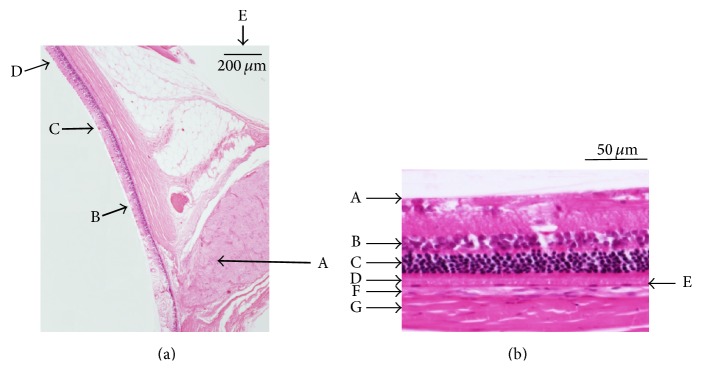
(a) The retina in a normal SD rat (hematoxylin and eosin stain). A: optic nerve disc. B–D: the points 500, 1,000, and 1,500 *μ*m from the optic nerve disc, respectively. E: index bar = 200 *μ*m. (b) The retina and choroid 400 to 600 *μ*m from the optic nerve disc. A: ILM. B: INL. C: ONL. D: PL. E: RPE. F: choroid. G: sclera.

**Figure 7:**
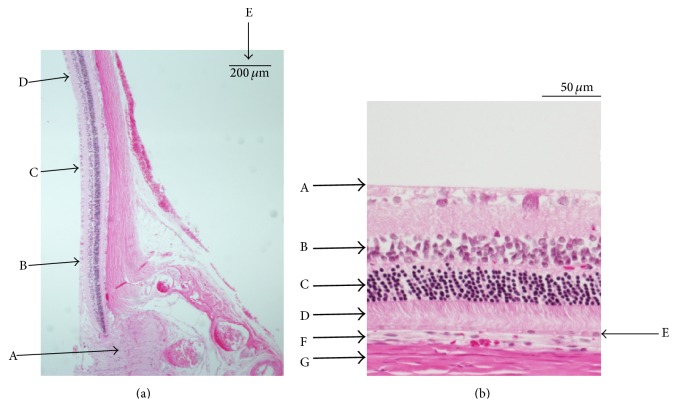
(a) The retina in a SDT rat (hematoxylin and eosin stain). A: optic nerve disc. B–D: the points 500, 1,000, and 1,500 *μ*m from the optic nerve disc, respectively. E: index bar = 200 *μ*m. (b) The retina and choroid 400 to 600 *μ*m from the optic nerve disc. A: ILM. B: INL. C: ONL. D: PL. E: RPE. F: choroid. G: sclera.

**Figure 8:**
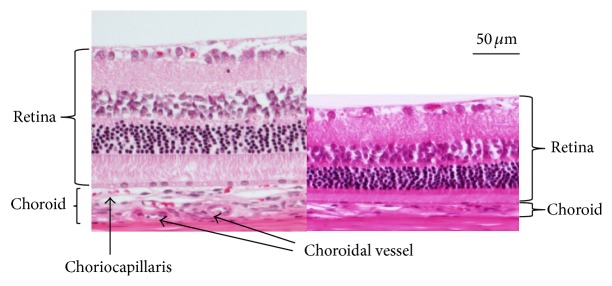
Comparison of the retina and the choroid in a SDT rat (left) and a normal SD rat (right) 1,400 *μ*m to 1,600 *μ*m from the optic nerve disc.

Figures 6, 7, and 8 show the retinas and choroids of a SDT rat and normal SD rat (hematoxylin and eosin stain). Compared with the normal SD rat, the retina and choroid in the SDT rat were thicker. Intense edema was present from the ILM to around the ganglion cells in the SDT rat. The intercellular space was much less dense in the SDT rat than in the SD rat throughout the retina and choroid. Two types of vessels were distinct in the choroid of the SDT rat. One, which was outside the RPE, was thought to be the choriocapillaris, and the other was thought to be a choroidal vessel. The walls of both the choriocapillaris and choroidal vessel were thickened diffusely. These findings were not detected in the normal SD rat.

## 4. Discussion 

In the current study, we evaluated retinal and choroidal edema in SDT rats. Large variations in the retinal thicknesses in both the normal SD and SDT rats in this experiment were seen and may present individual variations. A previous paper reported that at about 70 weeks of age SDT rats had PDR, the pathologic feature characterized by fibrous proliferation around the optic nerve disc [6]. Most of the SDT rats in this experiment were younger than 60 weeks of age, and the proliferative changes may appear about 10 weeks later. Retinal hemorrhage, exudates, and infiltration of inflammatory cells, which suggests typical nonproliferative diabetic retinopathy (NPDR), were not found in this experiment. The blood glucose level was extremely high and there was no macular formation in the SDT rat. Metabolic abnormalities and retinal morphology in the SDT rat differ from those in humans. Therefore, although DR in SDT rat mimics retinopathy in humans, to be precise it differs from that in humans. We reported accumulation of vascular endothelial growth factor (VEGF) and extensive fluorescein leakage around the optic nerve disc in the retinas of SDT rats [12], and we believe that the thickening in SDT rats is due to increasing retinal vascular permeability. Therefore, although typical NPDR in this experiment was not observed, we evaluated earlier DR as diabetic retinal edema in this experiment. Because the number of animals was limited in this experiment, we did not measure the retinal and choroidal thicknesses in SDT rats before the onset of diabetes. However, we reported that ranirestat suppressed the retinal thickness in SDT rats and the difference in the retinal thickness between the ranirestat-treated SDT rats and the normal SD rats was small [7]. Therefore, the retinal thickness in SDT rats may increase gradually after the onset of diabetes. Use of a polarizing microscope in the current study allowed us to observe the ILM, ganglion cells, INL, ONL, PL, and RPE in the retina; however, we could not distinguish the nerve fiber layer, the inner plexiform layer, or the outer plexiform layer from the other layers. A transmission electron microscope may be needed to examine more detailed pathologic features than what were apparent in this experiment. Edema was seen in each retinal section; no serous retinal detachment or cystoid edema was found in the SDT rats. This differs from DME in humans. The choroid in the SDT rats was significantly thicker than in the normal SD rats in this experiment. We are unaware of any study that mentioned the choroidal thickness in diabetic model rats, but some studies have addressed it in patients with DME using enhanced depth imaging spectral-domain-OCT. Unsal et al. [8] reported that the choroidal thicknesses in patients with PDR and DME decreased compared with healthy individuals. However, in that study, the patients with PDR had a history of treatment with panretinal laser photocoagulation (PRP). Kim et al. [10] reported that the choroidal thickness increased significantly as the diabetes progressed in severity from moderate-severe non-PDR to untreated PDR. However, in that study, the choroid in patients with PDR who had undergone PRP was thinner than that of patients with PDR who had not had any laser therapy. Although it is important to examine patients with DR under the same conditions, that is, durations of diabetes and DR, long-term glycemic control, age, and previous ocular treatment, it is difficult. Patients with diabetes often do not know definitively when the diabetes and DR developed, and the glycemic control of each patient varies. Therefore, determining whether the choroid is thick or thin in patients with DME may not be determined easily. The choroidal thickness in the SDT rats, which have almost the same conditions, is significantly thicker than in normal SD rats, but this result should not be simply extrapolated to patients with DME. SDT rats have no macula, and the retinal edema in SDT rats differs from DME in patients with diabetes. The choriocapillaris and choroidal vessels were distinct in the SDT rats, and the walls of both structures showed diffuse thickening. These findings suggested that a long duration of high blood glucose may affect the choroidal structure in SDT rats, but further transmission electron microscopy examinations are needed. We reported accumulation of VEGF and extensive fluorescein leakage around the optic nerve disc in the retinas of SDT rats [12]. Therefore, we suppose that the retinal edema results from increasing vascular permeability. However, we cannot explain why the choroidal edema appeared clearly in SDT rats in the current study. However, we believe that SDT rats are an ideal model of both diabetic retinal and choroidal edema. This rat model is expected to be used for investigating both DR and choroidopathy in many institutions.

## Figures and Tables

**Table 1:** The mean retinal and choroidal thicknesses in the normal SD rats. Each value is based on the distance from the optic nerve disc (500, 1,000, and 1,500 *µ*m). The average of the three values is calculated.

	500 *µ*m	1,000 *µ*m	1,500 *µ*m	Average
Total retinal thickness (*µ*m)	100.8 ± 17.7	96.6 ± 22.3	102.0 ± 26.5	99.8 ± 18.4
Thickness between ILM and INL (*µ*m)	47.5 ± 12.5	46.5 ± 11.7	50.0 ± 10.4	48.0 ± 7.9
INL thickness (*µ*m)	17.4 ± 5.4	16.8 ± 6.0	15.6 ± 5.9	16.6 ± 5.0
ONL thickness (*µ*m)	18.4 ± 3.9	19.0 ± 4.5	18.5 ± 5.0	18.7 ± 3.8
PL thickness (*µ*m)	10.5 ± 2.3	10.1 ± 3.0	9.5 ± 3.5	10.0 ± 2.6
Choroidal thickness (*µ*m)	8.8 ± 3.4	9.1 ± 3.9	9.5 ± 4.6	9.1 ± 3.0

**Table 2:** The mean retinal and choroidal thicknesses in the SDT rats. Each value is based on the distance from the optic nerve disc (500, 1,000, and 1,500 *µ*m). The average of the three values is calculated.

	500 *µ*m	1,000 *µ*m	1,500 *µ*m	Average
Total retinal thickness (*µ*m)	159.7 ± 29.4^*∗∗*^	146.6 ± 29.2^*∗∗*^	152.7 ± 33.6^*∗∗*^	153.0 ± 27.6^*∗∗*^
Thickness between ILM and INL (*µ*m)	78.8 ± 34.0^*∗*^	63.8 ± 17.3^*∗*^	68.8 ± 20.2^*∗*^	70.5 ± 18.8^*∗∗*^
INL thickness (*µ*m)	23.2 ± 4.1	25.2 ± 6.8^*∗*^	25.6 ± 7.5^*∗*^	24.7 ± 5.1^*∗∗*^
ONL thickness (*µ*m)	31.0 ± 6.9^*∗∗*^	33.2 ± 5.7^*∗∗*^	30.4 ± 7.3^*∗∗*^	31.5 ± 4.8^*∗∗*^
PL thickness (*µ*m)	21.7 ± 5.3^*∗∗*^	21.8 ± 5.8^*∗∗*^	22.5 ± 8.8^*∗∗*^	22.0 ± 5.4^*∗∗*^
Choroidal thickness (*µ*m)	12.8 ± 3.6	18.5 ± 4.1^*∗∗*^	21.8 ± 10.0^*∗∗*^	17.7 ± 5.4^*∗∗*^

Most layers are significantly thicker than in the normal SD rats, but there is no significant difference in the INL and choroidal thicknesses at 500 *µ*m.

^*∗*^ *P* < 0.05 compared with the normal SD rats.

^*∗∗*^ *P* < 0.01 compared with the normal SD rats.
